# Expression of USP18 and IL2RA Is Increased in Individuals Receiving Latent Tuberculosis Treatment with Isoniazid

**DOI:** 10.1155/2019/1297131

**Published:** 2019-12-06

**Authors:** Eleane de Oyarzabal, Lourdes García-García, Claudia Rangel-Escareño, Leticia Ferreyra-Reyes, Lorena Orozco, María Teresa Herrera, Claudia Carranza, Eduardo Sada, Esmeralda Juárez, Alfredo Ponce-de-León, José Sifuentes-Osornio, Robert J. Wilkinson, Martha Torres

**Affiliations:** ^1^Departamento de Microbiología, Instituto Nacional de Enfermedades Respiratorias Ismael Cosio Villegas, Ciudad de México, Mexico; ^2^Centro de Investigación sobre Enfermedades Infecciosas, Instituto Nacional de Salud Pública, Cuernavaca, Mexico; ^3^Computational and Integrative Genomics Laboratory, Instituto Nacional de Medicina Genómica (INMEGEN), Ciudad de México, Mexico; ^4^Laboratorio de Microbiología, Instituto Nacional de Ciencias Médicas y de Nutrición Salvador Zubirán, Ciudad de México, Mexico; ^5^Dirección Médica, Instituto Nacional de Ciencias Médicas y de Nutrición Salvador Zubirán, Ciudad de México, Mexico; ^6^Department of Medicine, Imperial College, Norfolk Place, London W2 1PG, UK; ^7^Wellcome Center for Infectious Diseases Research in Africa, Institute of Infectious Diseases and Molecular Medicine, Faculty of Health Sciences, University of Cape Town, Observatory 7925, South Africa; ^8^The Francis Crick Institute, London NW1 IAT, UK

## Abstract

**Background:**

The treatment of latent tuberculosis infection (LTBI) in individuals at risk of reactivation is essential for tuberculosis control. However, blood biomarkers associated with LTBI treatment have not been identified.

**Methods:**

Blood samples from tuberculin skin test (TST) reactive individuals were collected before and after one and six months of isoniazid (INH) therapy. Peripheral mononuclear cells (PBMC) were isolated, and an in-house interferon-*γ* release assay (IGRA) was performed. Expression of chemokine ligand 4 (CCL4), chemokine ligand 10 (CXCL10), chemokine ligand 11 (CXCL11), interferon alpha (IFNA), radical S-adenosyl methionine domain-containing 2 (RSAD2), ubiquitin-specific peptidase 18 (USP18), interferon-induced protein 44 (IFI44), interferon-induced protein 44 like (IFI44L), interferon-induced protein tetratricopeptide repeats 1(IFIT1), and interleukin 2 receptor subunit alpha (IL2RA) mRNA levels were assessed by qPCR before, during, and after INH treatment.

**Results:**

We observed significantly lower relative abundances of USP18, IFI44L, IFNA, and IL2RA transcripts in PBMC from IGRA-positive individuals compared to levels in IGRA-negative individuals before INH therapy. Also, relative abundance of CXCL11 was significantly lower in IGRA-positive than in IGRA-negative individuals before and after one month of INH therapy. However, the relative abundance of CCL4, CXCL10, and CXCL11 mRNA was significantly decreased and that of IL2RA and USP18 significantly increased after INH therapy, regardless of the IGRA result. Our results show that USP18, IFI44L, IFIT1, and IL2RA relative abundances increased significantly, meanwhile the relative abundance of CCL4, CXCL11, and IFNA decreased significantly after six months of INH therapy in TST-positive individuals.

**Conclusions:**

Changes in the profiles of USP18, IL2RA, IFNA, CCL4, and CXCL11 expressions during INH treatment in TST-positive individuals, regardless of IGRA status, are potential tools for monitoring latent tuberculosis treatment.

## 1. Introduction

Despite substantial efforts, tuberculosis (TB) remains a significant health care problem worldwide, as TB is one of the top 10 causes of death and the leading cause of infection by a single infectious agent [1]. Of the 1.7 billion people infected with *Mycobacterium tuberculosis* (*M*. *tuberculosis*) worldwide, a relatively small proportion (5-10%) develop TB, and in most cases, the disease occurs within 2 years [2]. Among the remaining infected people, the immune system can control the infection, but fails to eliminate the bacteria. Patients with this condition lack clinical symptoms, and the isolation of the bacteria from infected individuals is not feasible. This condition is called latent tuberculosis infection (LTBI) and provides a reservoir for future active TB cases [3]. LTBI is considered a state of a persistent immune response to stimulation with *M*. *tuberculosis* antigens with no evidence of clinically active TB [3]. The immunological factors involved in the host immune response during LTBI and its reactivation have not been completely elucidated. However, LTBI can persist throughout a person's lifetime, and factors that reduce immunity, such as human immunodeficiency virus (HIV) infection, antitumor necrosis factor treatment, corticosteroid medication, chemotherapy, and diabetes increase the risk of developing active TB [4, 5]. Therefore, individuals with an increased risk of progression to active TB disease would benefit from testing and treatment of LTBI [6].

Although a gold standard test for LTBI is not available, two methods are used to infer LTBI: the tuberculin skin test (TST) and interferon-*γ* release assays (IGRA). The TST was the only screening tool for LTBI for over a century [7], though one of the major disadvantages of the TST is its lack of specificity, particularly in populations vaccinated with bacille Calmette-Guérin (BCG) [8]. IGRAs, including the QuantiFERON-TB test (QFT) and the T-SPOT.TB test, use the early secretory antigen-6 (ESAT-6) and culture filtrate protein-10 (CFP-10) proteins that are present in *M*. *tuberculosis* but not in most nontuberculous mycobacteria, particularly BCG [9]. The use of the TST and IGRA in a two-step approach has revealed discrepancies between the two tests [10]. Although most discordant results have been detected in the BCG-vaccinated population, in some cases, the discordance occurs independently of prior BCG vaccination [11].

The decision to perform a test for LTBI is accompanied by the intention to treat because the goal of LTBI treatment is to decrease the risk of developing active TB disease [6, 12]. The guidelines for LTBI treatment recommend that isoniazid (INH) should be administered to both adults and children daily at a dose of 5 mg/kg with a maximum dose of 300 mg/day over 6 months in countries with high and low TB incidence rates [3]. Recently, new schemes for LTBI treatment in children and adults from countries with high and low incidence rates of TB have been recommended, including rifampicin plus INH or rifapentine plus INH for three months. Additionally, the new recommendation includes an individualized risk assessment for preventive treatment of high-risk household contacts among patients with multidrug-resistant TB [3]. Several studies have reported changes in *M*. *tuberculosis*-specific T-cell responses during preventive therapy; however, the results are inconsistent [13–15]. Additionally, neither IFN*γ* levels nor the number of IFN*γ*-producing cells detected after INH treatment can be reliably associated with LTBI treatment [16–18]. Therefore, despite the efficacy of INH treatment, correlates indicating an effective response to LTBI treatment are lacking. Nonetheless, blood-based markers would be useful for both evaluating new regimens and developing of new treatments. Hence, to identify the treatment-associated transcript profile, we selected IFN*α*-inducible genes previously reported in active TB [19, 20] and genes assessed in LTBI and in models of infection [21–23] and during treatment of TB [24–27] and evaluated the expression profile of chemokine ligand 4 (CCL4), chemokine ligand 10 (CXCL10), chemokine ligand 11 (CXCL11), interferon alpha (IFNA), radical S-adenosyl methionine domain-containing 2 (RSAD2), ubiquitin-specific peptidase 18 (USP18), interferon-induced protein 44 (IFI44), interferon-induced protein 44 like (IFI44L), interferon-induced protein tetratricopeptide repeats 1(IFIT1), and interleukin 2 receptor subunit alpha (IL2RA) mRNAs in peripheral blood mononuclear cells (PBMC) from individuals with LTBI before, during, and after INH treatment. We found that changes in the expression of USP18, IL2RA, IFNA, CCL4, and CXCL11 during and after INH treatment represent a potentially useful tool for monitoring LTBI treatment.

## 2. Materials and Methods

### 2.1. Study Population and Inclusion Criteria

From 2008-2009, we recruited healthy, asymptomatic volunteers (*n* = 280) who were in close contact with patients with pulmonary TB, without clinical or radiological evidence of TB disease, aged 21 years or older, and reactive to TST (>10 mm) from reference health centers in Orizaba, Veracruz, México. The TB incidence of this region ranked among the ten highest in Mexico in 2016, with 27.4 cases per 100,000 inhabitants [28].

**Figure 1:**
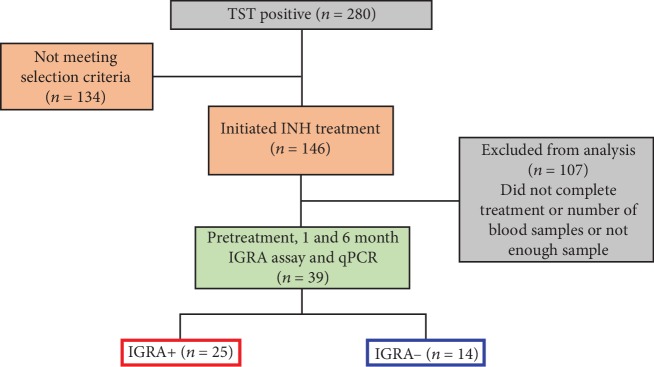
Flow diagram of the study population.

We excluded volunteers who were pregnant, immunosuppressed, HIV-positive, TST-negative, or diagnosed with other clinical conditions (*n* = 134). The participants received INH (5 mg per kg of body weight, up to 300 mg per day, for 6 months). We also excluded volunteers who did not complete treatment or refused to donate all samples or whose samples were insufficient (*n* = 107, Figure 1). Participants underwent clinical evaluation. Adherence was periodically monitored by measurement of INH metabolites in urine using Taxo-INH strips (Becton Dickinson, Sparks, MD, USA) and pill counting. Ten milliliters of blood was obtained for PBMC isolation at the following time points: pretreatment (t0), at one month (t1), and six months (t6) after the initiation of treatment. The blood samples were applied to a Lymphoprep (Axis-Shield PoC AS, Oslo, Norway) gradient and centrifuged to obtain PBMC. These cells were used for IGRA and preserved at -70°C until the qPCR assay was performed. Based on the results of the in-house IGRA, the participants were subclassified into IGRA-positive and IGRA-negative groups.

All assays were performed by personnel blinded to the participant's status. Ethical approval for the protocol was obtained from the Ethics Committees of the Instituto Nacional de Enfermedades Respiratorias Ismael Cosío Villlegas and the Instituto Nacional de Salud Pública, Mexico, and written informed consent was obtained from all participants. The study was registered at https://ClinicalTrials.gov under NCT00293228.

### 2.2. LTBI

LTBI was defined by the reactivity of TST (>10 mm), and individuals were subclassified according to the in-house IGRA result for ESAT-6, CFP10, or both antigens.

### 2.3. TST

This test was performed using 0.1 ml (5 tuberculin units, UT) of tuberculin purified protein derivative (PPD) (RT-23; Statens Serum Institut, Copenhagen, Denmark). The result was evaluated at 48 h post-injection. A positive or reactive TST indicated TB infection and was defined as ≥10 mm diameter of induration according to the official Mexican policy for the prevention and control of TB (NOM-006-SSA2-1993) (http://www.salud.gob.mx/unidades/cdi/nom/006ssa23.html).

### 2.4. IGRA

The in-house IGRA was performed as previously described [29]. Briefly, PBMC were obtained from heparinized peripheral blood and resuspended in RPMI 1640 culture medium containing 2 mM L-glutamine (BioWhittaker, Radnor, PA, USA). The cells were counted using the trypan blue exclusion method, and cell viability was typically close to 93%. PBMC were plated in flat-bottomed, 96-well culture plates at a density of 2 × 10^5^ cells per well and incubated with custom-designed overlapping synthetic peptides covering the protein sequences of the antigens ESAT-6 or CFP-10 (5 *μ*g/ml, Peptide Synthetic UK and ProImmune, Oxford, UK), PPD (10 *μ*g/ml, Statens Serum Institute,), phytohemagglutinin (PHA) (10 *μ*g/ml, Sigma-Aldrich, St. Louis, MO, USA), and culture medium. After 6 days of cultivation at 37°C in a 5% CO_2_ atmosphere, the cell-free supernatants were collected, and IFN*γ* secretion was analyzed by ELISA. The detection limit of the IFN*γ* ELISA was 8 pg/ml. ESAT-6- or CFP10-specific IFN*γ* responses were considered positive when the values were > 100 pg/ml after subtracting the values obtained from unstimulated cells, as previously described [29]. A value of >100 pg/ml was defined as three-fold higher than the median (34 pg/ml) of unstimulated cells.

### 2.5. Real-Time Quantitative PCR (qPCR)

qPCR was used to evaluate the effect of INH therapy on the expression of selected genes (Table 1) [19–27]. Total RNA of 1 × 10^6^ PBMC from individuals with LTBI before and after INH therapy was extracted with the RNeasy Mini Kit (Qiagen, CA, USA), and cDNAs were synthesized using a Superscript First-Strand set (Invitrogen, CA, USA). TaqMan Gene Expression Assays (Applied Biosystems, CA, USA), the TaqMan Universal Master Mix (Applied Biosystems, NJ, USA), and TaqMan probes for IFIT1 (Hs03027069_s1), IFI44 (Hs00951349_m1), IFI44L (Hs00915292_m1), IFNA (Hs003406429_gH), CXCL10 (Hs01124252_g1), CXCL11 (Hs04187682_g1), USP18 (Hs00276441_m1), IL2RA (Hs00907778_m1), RSAD2 (Hs00369813_m1), and CCL4 (Hs04421399_gH) and the endogenous control ribosomal 18S RNA(Hs999999001_s1) were used for qPCR. A StepOnePlus Real-Time PCR Systems (Applied Biosystems) was used to amplify the genetic material. Expression values for target genes were normalized to 18S expression, Ct values from all samples were obtained with the same software, and relative gene expression analysis was performed using the delta Ct method (2^-*ΔΔ*Ct^). The mRNA relative abundance was calculated as the Ct relative to 18S expression.

### 2.6. Statistical Analysis

GraphPad Prism software version 6.0 for Mac (GraphPad Software, San Diego, CA, USA) was used for statistical analyses. The details of the statistical tests are provided in the figure legends.

## 3. Results

### 3.1. Recruitment of Individuals with LTBI

We included thirty-nine participants who completed six months of INH treatment (5 mg/kg daily), as depicted in Figure 1. All individuals had a positive TST result averaging 12.8 mm.  Table 2 shows the demographic data of the participants. The majority of the population (60.5%) was female. The average age was 39.2 years. The participants were divided into two groups based on the results of the in-house IGRA response to ESAT-6 or CFP-10 or both overlapping peptides as follows: IGRA-positive (*n* = 25) and IGRA-negative (*n* = 14) (Table 2).

### 3.2. Differentially Expressed Genes in IGRA-Positive and IGRA-Negative Individuals before INH Therapy Assessed by qPCR

**Figure 2:**
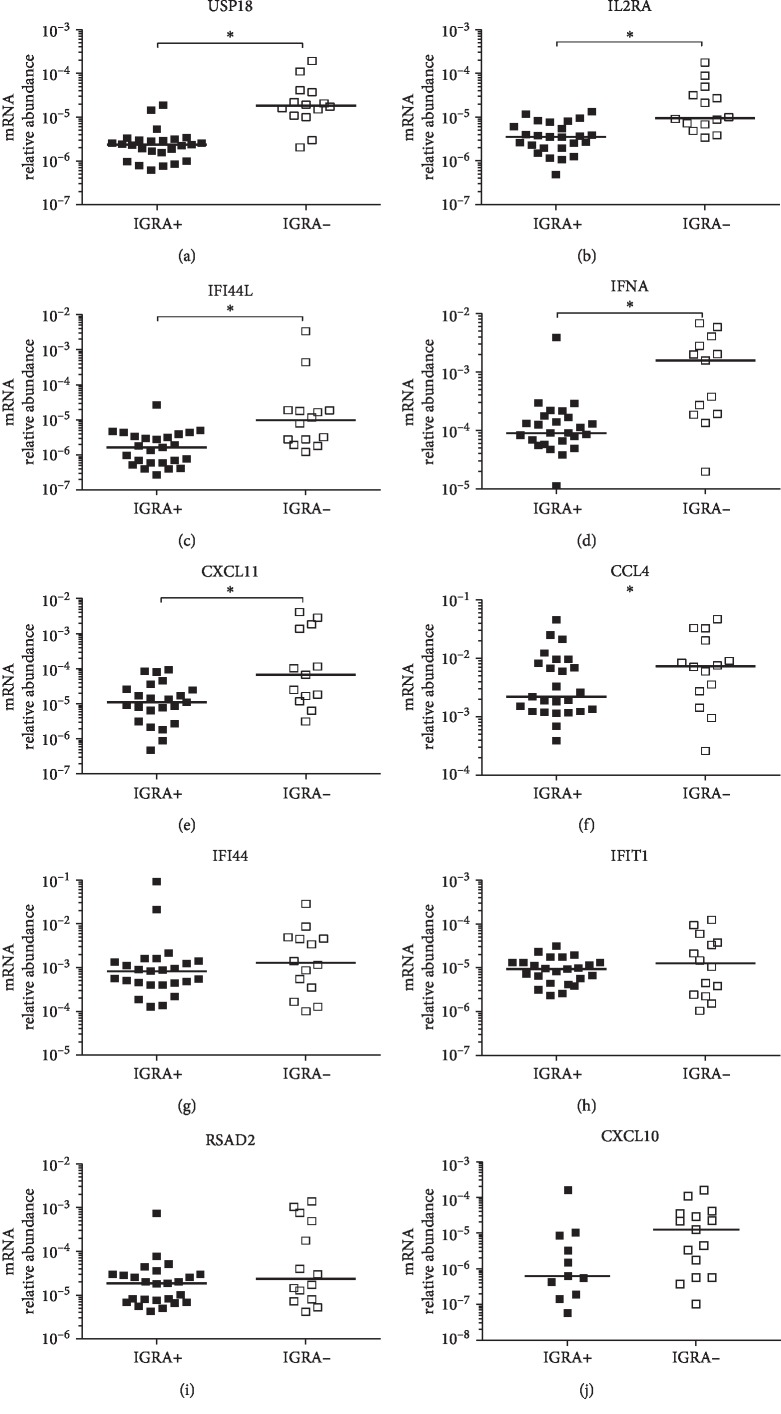
Relative levels of the CXCL11, IFNA, RSAD2, USP18, IFI44, IFI44L, IFITI, CCL4, and IL2RA mRNAs in PBMC from IGRA-positive (*n* = 25, black square) or IGRA-negative (*n* = 14, open square) individuals before INH treatment. Gene expression was determined by real-time qPCR. Values are presented as levels relative to 18S RNA. Individual results are displayed, and the line indicates the median. ^∗^ *p* < 0.05, Mann-Whitney *U* test.

We first investigated whether there were differences in gene expression associated with IGRA status. Therefore, we determined the relative abundances of CCL4, CXCL10, CXCL11, IFNA, RSAD2, USP18, IFI44, IFI44L, IFIT1, and IL2RA transcripts in PBMC from IGRA-positive (*n* = 25) and IGRA-negative (*n* = 14) individuals before INH therapy. We observed significantly lower relative abundances of USP18, IL2RA, IFI44L, IFNA, and CXCL11 mRNAs among the IGRA-positive individuals compared to IGRA-negative individuals before INH therapy (t0) (Figures 2(a)–2(e)). Conversely, no significant differences in the relative abundances of the CCL4, IFI44, IFIT1, and RSAD2 mRNAs were observed between the IGRA-positive and IGRA-negative individuals before INH therapy (Figures 2(f)–2(i)). Interestingly, no significant difference in the relative abundance of CXCL10 mRNA was observed either; however, only 44% of IGRA-positive individuals expressed detectable levels of CXCL10 mRNA, whereas 93.75% of IGRA-negative individuals expressed this chemokine before INH therapy (Figure 2(j)).

### 3.3. Differentially Expressed Genes in IGRA-Positive and IGRA-Negative Individuals after One and Six Months of INH Therapy Assessed by qPCR

**Figure 3:**
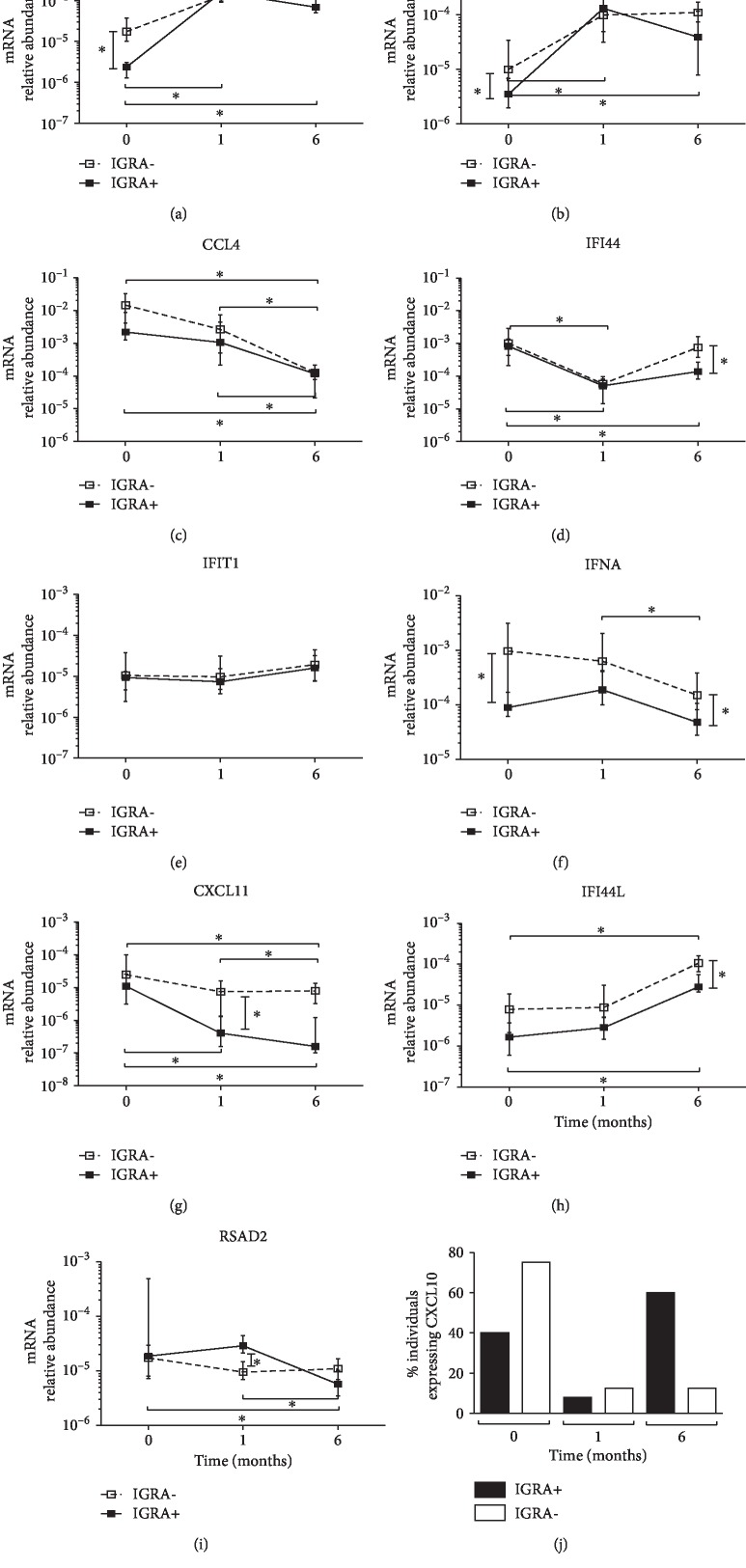
CXCL11, IFNA, RSAD2, USP18, IFI44, IFI44L, IFITI, CCL4, and IL2RA expressions in PBMC from IGRA-positive individuals (*n* = 25, black square) or IGRA-negative (*n* = 14, open square) individuals before and after one and six months of INH treatment. Gene expression was assessed by qPCR. (a–i) The median interquartile range of gene expression relative to that of 18S RNA after one and six months of treatment is reported. The statistical analysis was performed using the nonparametric Kruskal-Wallis ANOVA followed by Dunn's test; ∗*p* < 0.05. (j) CXCL10 expression detected in PBMC from IGRA-positive individuals (*n* = 11) and IGRA-negative individuals (*n* = 12) before and after one and six months of INH treatment is presented as the percentage of individuals expressing the gene.

We used qPCR to analyze the relative abundance of CCL4, CXCL10, CXCL11, IFNA, RSAD2, USP18, IFI44, IFI44L, IFIT1, and IL2RA mRNAs in PBMC from IGRA-positive and IGRA-negative individuals before initiation and after one and six months of INH therapy. USP18 and IL2RA expressions significantly increased in both IGRA-positive and IGRA-negative individuals after one and six months of INH therapy compared to the levels before INH therapy (Figures 3(a) and 3(b)). In contrast, CCL4 expression significantly decreased among all individuals regardless of their IGRA status after six months of INH therapy (Figure 3(c)). IFI44 expression significantly decreased among all individuals after one month of INH therapy (Figure 3(d)). Expression of IFIT1 did not show significant changes after INH therapy (Figure 3(f)). Interestingly, IFNA expression was significantly higher in IGRA-negative individuals than in IGRA-positive individuals before INH therapy, and this difference was also significant after six months of INH therapy (Figure 3(e)). CXCL11 expression was decreased in both IGRA-positive and IGRA-negative individuals after six months of INH therapy, but the decrease was significantly higher among the IGRA-positive individuals after one month of INH therapy (Figure 3(g)). Strikingly, CXCL10 expression dynamics were challenging to follow in the same manner because the percentage of individuals of both groups expressing this chemokine diminished dramatically after one month of treatment, and only IGRA-positive individuals showed increased CXCL10 expression after six months of treatment (Figure 3(j)).

### 3.4. qPCR Analysis of the 10 Selected Genes in TST-Positive Individuals after INH Therapy

**Figure 4:**
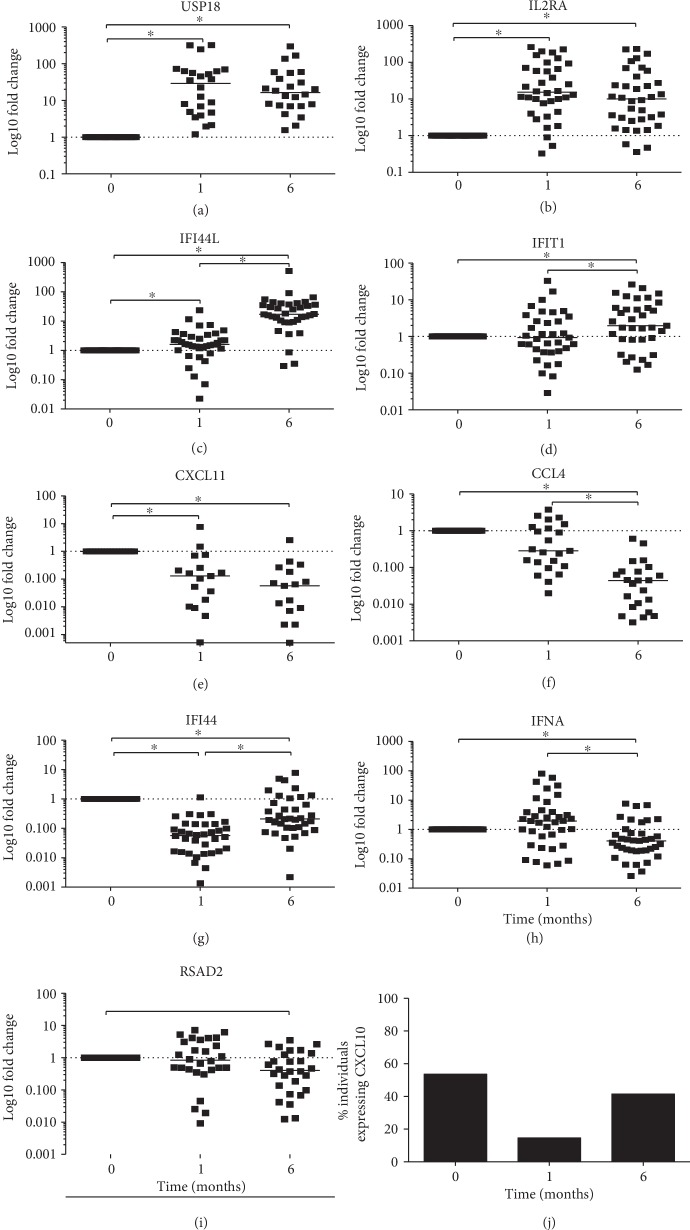
CXCL11, IFNA, RSAD2, USP18, IFI44, IFI44L, IFITI, CCL4, and IL2RA mRNA expressions before and after one and six months of INH treatment in TST-positive individuals. (a–i) The fold changes after one and six months are reported relative to the expression levels prior to INH treatment (t0) using the comparative delta Ct method (*n* = 39). Individual results are displayed, and the line indicates the median. statistical analysis was performed using non-parametric Friedman's ANOVA followed by Dunn's test; ^∗^ *p* < 0.05. (j) CXCL10 expression detected in PBMC from TST-positive individuals (*n* = 39) before and one and six months after INH treatment is presented as the percentage of individuals expressing the gene.

Because several genes were consistently modified following INH treatment regardless of the IGRA status and taking into consideration that TST positivity is the only indicator of LTBI in some individuals, we examined the fold change in gene expression of the above genes after one and six months of INH treatment in all individuals relative to t0 (Figure 4 and Table 3). USP18, IL2RA, and IFI44L expression significantly increased among all participants after one month of INH therapy (Figures 4(a)–4(c)). In contrast, CXCL11 and IFI44 expressions decreased significantly during the same time frame (Figures 4(e) and 4(g)).

USP18, IL2RA, IFI44L, and IFIT1 expressions increased significantly after six months of INH therapy; however, only the increase in IFI44L, IFI44, and IFIT1 was significant in comparison with levels at one month of INH treatment (Figures 4(a)–4(d)).

Conversely, CXCL11, CCL4, IFI44, IFNA, and RSAD2 expressions decreased significantly after six months of treatment, and CCL4 and IFNA expression also showed a significant decrease in comparison to one month of INH therapy (Figures 4(e)–4(i) and Table 3). Overall, USP18 and IL2RA expressions significantly increased after one and six months of INH therapy (Figures 4(a) and 4(b)). Also, CXCL10 expression decreased to undetectable levels after one month of INH therapy in most of the participants in comparison to before treatment (Figure 4(j)).

## 4. Discussion

Few studies have assessed the effect of INH therapy on the immune response in blood cells from individuals with LTBI [13–16]. In the present study, we defined LTBI based on a positive result of the TST. Among the participants, 64% were IGRA-positive as determined by long-incubation in-house IGRA that allowed us to detect IFN*γ* from both effector memory cells and central memory T-cells, both of which are involved in TB infection [30]. Although TST specificity is limited among BCG-vaccinated populations, and neither test predicts subsequent development of active TB among household contacts of pulmonary TB patients. However, TST remains the preferred method for LTBI diagnosis in resource-limited, high TB burden settings due to its low cost and straightforward implementation.

Since our population was vaccinated at birth, and the mean age was 39.2 years, BCG cross-reactivity is unlikely to explain the discordance observed in the TST-positive individuals who are IGRA negative. In addition, it has been reported that vaccination in infancy has minimal effect on TST results after 10 or more years of vaccination [31]. Different studies have shown the importance of interpreting both TST and IGRA results in the context of prevalence and exposure [32–34]. We studied individuals who had been exposed to pulmonary tuberculosis patients and were, therefore, more likely infected. In addition, it was reported that household contacts that were TST positive but IGRA negative had less exposure to the index case compared to the concordant group [35]. These data suggest that following *M*. *tuberculosis* exposure, IGRA conversion may take longer than TST [35]. Therefore, TST-positive individuals with negative IGRA results as the ones that occurred in our study should not be interpreted as the absence of infection since previous studies in Mexican population and in high tuberculosis burden settings have shown that TST results correlates well with *M*. *tuberculosis* infection [36, 37].

When we analyzed the expression of the selected genes in individuals according to IGRA results before INH treatment, we found that expression of IFI44L, IFNA, IL2RA, and USP18 was significantly lower in IGRA-positive individuals than in IGRA-negative individuals. These results suggest that concerning the assessed genes before INH therapy, TST-reactive individuals harbor immunological differences according to their IGRA result.

Among other genes identified as relevant in our study, expression of CXCL10 (also known as IP-10), CXCL11, and CCL4 was significantly decreased after six months of INH therapy compared to baseline levels and after one month of treatment, regardless of the IGRA status. CXCL10 has been used to distinguish between TB and LTBI [26]. In addition, a decrease in plasma CXCL10 levels after two weeks of TB treatment has been reported [27]. Although the role of CXCL10 in LTBI treatment has not been studied, decreased CXCL10 expression after a few weeks of INH treatment may indicate reduced antigen load. CCL4 is overexpressed in lung tissues of mice with TB and in patients with late-stage TB [25], suggesting that the significant decrease in CCL4 expression observed in individuals with LTBI after INH treatment might be associated with a decreased risk of developing active TB and could, therefore, be useful as a biomarker of treatment efficacy.

USP18 and IL2RA expressions significantly increased after one and six months of INH therapy in both IGRA-negative and IGRA-positive individuals. These results suggest that INH therapy is not associated with a differential gene expression profile between IGRA-negative and IGRA-positive individuals, but rather with a general immunomodulatory effect of the drug. Therefore, upregulation of both genes might be a useful tool to monitor the response of INH therapy and might be associated with the efficacy of INH therapy. However, the evaluation of the efficacy of INH treatment was beyond the scope of this study.

Overall, the qPCR analysis revealed an association between INH treatment and significant changes in the expression of nine of the ten selected genes among TST-positive individuals. We hypothesize that these changes may be associated with the INH bactericidal effect.

Among TST-positive individuals, USP18, IL2RA, and CXCL11 expression levels changed significantly after one month of INH therapy, remaining without significant changes at six months of therapy. These initial change may be associated with an early INH bactericidal effect, and prolonged INH administration seems not to produce subsequent modification of expression levels of these genes. It is also possible that the time frame of our study was unable to assess the kinetics of the expression of these genes.

Additionally, we also observed that the expression of ISGs such as IFIT1, IFI44, and IFI44L increased significantly after six months in comparison to one month of INH treatment among TST-positive individuals. In this regard, it has been described that a variety of stimuli, such as infection with RNA or DNA viruses and viral and bacterial molecular patterns (PAMPs), directly induce transcription of the IFIT1/ISG56 family [38]. Therefore, mycobacterial antigens may be continuously released during INH treatment and induce the expression of these genes.

In contrast, IFNA expression was significantly decreased after six months in comparison to one month of INH therapy in TST-positive individuals. This result suggests that six months of INH treatment induced an increase in USP18 and IFI44L expressions that negatively regulated IFNA [39, 40] explaining its low levels after six months of INH therapy. So, our observation that there is an increase in the expression of genes that regulate IFNA after six months of INH treatment indicates that USP18 and IFI44L expressions might be relevant as biomarkers of the effectiveness of INH therapy. These results also support the suitability of prolonged INH administration.

In particular, IFNA expression decreased significantly in PBMC of TST-positive individuals after six months of INH therapy compared to the expression levels recorded before treatment and after one month of treatment. Because viral and bacterial persistence are associated with deleterious immunoregulatory effects of sustained type I IFN expression [41], this decrease in IFNA expression might be due to a reduction in the latent bacterial load caused by INH therapy. Therefore, we speculate that INH therapy kills the bacteria in LTBI and decreases the bacterial burden necessary to sustain IFNA expression. In addition, IFN*α* production is associated with suppression of the innate immune response [42, 43]. Thus, an increase in type I IFN signaling may lead to increased susceptibility of the host to bacterial infections [44, 45]. Type I IFN is also associated with TB susceptibility, as has been demonstrated by the observation that IFN*α* treatment in patients with hepatitis D infection exacerbates TB [46]. One of the most prevalent biosignatures in the blood of patients with TB is the elevated expression of transcripts involved in type I and type II IFN signaling, mainly driven by neutrophils [19]. Moreover, IFN*α* signaling has been proposed as a valuable predictive biomarker of the progression of LTBI toward active TB disease [47]. Accordingly, we suggest that the decrease in IFNA expression observed after INH therapy represents a potential biomarker of INH efficacy treatment in individuals with LTBI.

We also noted that the USP18 expression was increased in PBMC from TST-positive individuals after INH therapy. Such upregulation of USP18 expression and the decrease in IFNA expression after six months of INH therapy are consistent with the function of USP18, which is a potent negative regulator of IFNA signaling [40]. USP18 regulates the IFN type I signaling pathway by inhibiting IFN type I-induced JAK/STAT activation and removes interferon-stimulated genes 15 (ISG15) adducts from substrate proteins through its independent function as a protease [48]. USP18 might play a role in controlling bacterial infections, as suggested in a study using mice with a mutation in USP18 that showed increased bacterial loads, increased inflammatory responses, and increased activity of the type I IFN signaling pathway [21]. The mechanism regulating USP18 during INH therapy is unknown, but patients with a deficiency in ISG15 are susceptible to mycobacterial diseases, and the absence of intracellular ISG15 prevents the accumulation of USP18, resulting in amplification of IFN*α*/*β* responses [49]. Consequently, the upregulation of USP18 during INH therapy observed in the present study suggests that it has an essential role in controlling *M*. *tuberculosis* infection and may constitute a potential biomarker for LTBI treatment.

Additionally, expression of other interferon-stimulated genes (ISGs), such as IFIT1, increased significantly in PBMC from TST-positive individuals after six months of INH therapy. IFIT1 and RSAD2 expressions were recently shown to be induced in macrophages infected with *M*. *tuberculosis* [23]. Therefore, such an increase in the expression of these ISGs may be involved in the antimycobacterial activity of INH therapy, though, the precise mechanisms involved remain unknown and are worthy of exploration. Our results showed that the expression of IFI44L was significantly increased in PBMC from TST-positive individuals after six months of INH therapy. In contrast, other authors have reported that the expression of IFIT44L in patients with active TB is significantly reduced after six months of TB treatment [20].

IFI44 is an IFN*α*-inducible protein that is associated with several viral infections, but its role in bacterial infections is unknown [50]. IL2RA expression, which is associated with T-cell activation and regulatory cell profiles, was significantly increased in PBMC from TST-positive individuals after one and six months of INH therapy. In contrast, it has been reported that treatment of active TB is associated with significantly decreased levels of soluble IL2R after six months of anti-TB therapy for pleural TB, though these reduced levels are still higher than those in controls [51]. In addition, it has been reported that s-IL2R levels are higher in TST-positive individuals than in TST-negative controls [22].

Thus, changes in gene expression profiles associated with INH therapy in TST-positive individuals, regardless of the IGRA status, represent potentially useful tools to monitor the response to INH treatment and to evaluate new alternative LTBI regimens.

## 5. Limitations

The main limitation of our study is the unavailability of a hard-clinical endpoint of bacterial clearance in individuals with LTBI that prevented us from determining the association between differential expression of USP18, IL2RA, IFNA, CXCL11, or CCL4 mRNAs and the efficacy of INH therapy. Another limitation is that we did not control for intraindividual changes in gene expression over time.

In conclusion, in the present study, we observed increased USP18 and IL2RA expressions after one and six months of INH therapy and decreased IFNA, CCL4, and CXCL11 expressions after six months of LTBI treatment. These results might facilitate the use of mRNA expression as a tool for monitoring INH treatment and may have the potential for monitoring new alternative LTBI regimens.

## Figures and Tables

**Table 1:** Genes selected for qPCR analysis.

No	Gene	Name	Signaling pathway	Reported in TB	Reference
1	CCL-4	C-C motif chemokine ligand 4	Cytokine signaling in immune system, Toll-like receptor signaling pathway	Yes	[25]
2	CXCL-10	C-X-C motif chemokine ligand 10	Peptide ligand-binding receptors, Toll-like receptor signaling pathway, type II interferon signaling (IFNG)	Yes	[26, 27]
3	CXCL-11	C-X-C motif chemokine ligand 11	Peptide ligand-binding receptors, Toll-like receptor signaling pathway, type II interferon signaling (IFNG)	Yes	[24]
4	IFI44	Interferon-induced protein 44	Immune response IFN alpha/beta signaling pathway	Yes	[19]
5	IFI44L	Interferon-induced protein like 44	Immune response IFN alpha/beta signaling pathway	Yes	[19, 20]
6	IFIT1	Interferon-induced with tetraticopeptide repeats 1	Immune response IFN alpha/beta signaling pathway	Yes	[23]
7	IFNA	Interferon alpha 10	Immune response IFN alpha/beta signaling pathway	Yes	[19]
8	IL2RA	Interleukin 2 receptor subunit alpha	TGF-beta pathway, Th17 cell differentiation	Yes	[22]
9	RSAD2	Radical S-adenosyl methionine domain-containing 2	Immune response IFN alpha/beta signaling pathway	Yes	[23]
10	USP18	Ubiquitin-specific peptidase 18	Immune response IFN alpha/beta signaling pathway, antiviral mechanism by IFN-stimulated genes	Yes	[21]

**Table 2:** Demographic data of the study population.

	IGRA+	IGRA–	*p*
*n*	25	14	
Gender/women (%)	56.0 14/25	64.3 9/14	NS
Age (years)	39.9 ± 2.1	38.4 ± 3.4	NS
TST induration (mm)	13.1 ± 0.5	12.6 ± 0.6	NS
IFN*γ* production to ESAT-6 (pg/mL)	840 ± 351	4.7 ± 2.0	0.0033
IFN*γ* production to CFP-10 (pg/mL)	4779 ± 2798	17.7 ± 5.9	0.0063
IFN*γ* production to PPD (pg/mL)	19694 ± 3758	7246 ± 2543	0.0579
IFN*γ* production to PHA (pg/mL)	42816 ± 12169	26019 ± 6659	0.3167

**Table 3:** qPCR analysis of differentially expressed genes in PBMC from TST-positive individuals after 1 and 6 months of INH therapy.

No.	Gene	Comparison			^∗^ *p* ≤ 0.05
		t1 vs. t0	t6 vs. t0	t1 vs. t6	
1	USP18	Up	Up	—	^∗^a, b
2	IL2RA	Up	Up	—	^∗^a, b
3	IFI44L	Up	Up	Up	^∗^a, b, c
4	IFIT1	—	Up	Up	^∗^b, c
5	CXCL-11	Down	Down	—	^∗^b
6	CCL4	—	Down	Down	^∗^b, c
7	IFI44	Down	Down	Up	^∗^a, b, c
8	IFNA	—	Down	Down	^∗^b, c
9	RSAD2	—	Down	—	^∗^c
10	CXCL-10	—	—	—	NS

Mann-Whitney Test; NS: not significant; a = t1 vs. t0; b = t6 vs. t0; c = t1 vs. t6.

## Data Availability

The data used to support the findings of this study are included in supplementary information files ([Supplementary-material supplementary-material-1]).

## Supplementary Materials

Supplementary Materials Data set of IGRA-positive and IGRA-negative individuals, Time of sampling and qPCR data set for all the assessed genes.
